# Weight and metabolic effects of cpap in obstructive sleep apnea patients with obesity

**DOI:** 10.1186/1465-9921-12-80

**Published:** 2011-06-15

**Authors:** Jose M Garcia, Hossein Sharafkhaneh, Max Hirshkowitz, Rania Elkhatib, Amir Sharafkhaneh

**Affiliations:** 1 Division of Diabetes, Endocrinology and Metabolism, Michael E. DeBakey Veterans Affairs Medical Center, 2002 Holcombe Blvd., Houston, Texas, 77025, USA; 2 Division of Pulmonary, Critical Care and Sleep Medicine, Michael E. DeBakey Veterans Affairs Medical Center, 2002 Holcombe Blvd., Houston, Texas, 77025, USA; 3 Huffington Center on Aging, Baylor College of Medicine, One Baylor Plaza, Houston, TX, 77030, USA; 4 Department of Medicine, Section of Pulmonary, Critical Care and Sleep Medicine, Baylor College of Medicine, One Baylor Plaza, Houston, TX, 77030, USA

## Abstract

**Background:**

Obstructive sleep apnea (OSA) is associated with obesity, insulin resistance (IR) and diabetes. Continuous positive airway pressure (CPAP) rapidly mitigates OSA in obese subjects but its metabolic effects are not well-characterized. We postulated that CPAP will decrease IR, ghrelin and resistin and increase adiponectin levels in this setting.

**Methods:**

In a pre- and post-treatment, within-subject design, insulin and appetite-regulating hormones were assayed in 20 obese subjects with OSA before and after 6 months of CPAP use. Primary outcome measures included glucose, insulin, and IR levels. Other measures included ghrelin, leptin, adiponectin and resistin levels. Body weight change were recorded and used to examine the relationship between glucose regulation and appetite-regulating hormones.

**Results:**

CPAP effectively improved hypoxia. However, subjects had increased insulin and IR. Fasting ghrelin decreased significantly while leptin, adiponectin and resistin remained unchanged. Forty percent of patients gained weight significantly. Changes in body weight directly correlated with changes in insulin and IR. Ghrelin changes inversely correlated with changes in IR but did not change as a function of weight.

**Conclusions:**

Weight change rather than elimination of hypoxia modulated alterations in IR in obese patients with OSA during the first six months of CPAP therapy.

## Background

Obstructive sleep apnea (OSA) is characterized by sleep-related airway obstructions that produce apnea. These events provoke arousals and cause oxygen desaturations and heightened sympathetic activity during sleep and waking hours [1] that may play a role in the development of insulin resistance [2]. Obesity is a strong risk factor for OSA [3] and both obesity and OSA are associated with increased insulin resistance and diabetes [4].

Hormones involved in the regulation of body weight and glucose metabolism include ghrelin, leptin, adiponectin and resistin. Ghrelin is an orexigenic hormone and it has been proposed as a cause of increased appetite and obesity [5]. Administration of ghrelin increases adiposity, food intake and body weight [6]. It also regulates glucose homeostasis increasing glucose levels and decreasing insulin secretion [7]. Leptin is a hormone secreted by adipocytes in proportion to fat mass. It is elevated in obesity and its administration suppresses appetite and induces weight loss [8]. Resistin and adiponectin are also adipocyte-derived hormones linked to obesity, insulin resistance, and diabetes. Adiponectin levels inversely correlate with BMI and are lower in individuals with diabetes whereas resistin directly correlates with obesity and insulin resistance.

Whether treatment of OSA can reverse insulin resistance and prevent body weight gain is controversial. Because hypoxemia-induced sympathetic activation is thought to be the source of the endocrine abnormalities often seen in patients with OSA, and continuous positive airway pressure (CPAP) effectively reverses hypoxemia in patients with OSA, we hypothesized that CPAP will decrease insulin resistance, ghrelin and resistin levels and increase adiponectin levels in a group of obese individuals with OSA.

## Methods

### Study design and experimental subjects

The protocol was approved by the Baylor College of Medicine Institutional Review Board, and the Research and Development Committee of the Michael E. DeBakey Veterans Affairs Medical Center in Houston, Texas. This study was conducted between April 2004 and March 2006. All clinical investigation was conducted in accordance with the guidelines in The Declaration of Helsinki and all subjects provided written informed consent.

Adult subjects with no prior history of diabetes were recruited from patients referred to the hospital's Sleep Center for evaluation of OSA. OSA was confirmed by laboratory polysomnography (PSG). Twenty-three patients with an apnea+hypopnea index (AHI) ≥15 obstructive and/or mixed events/hour as criteria participated in the project. We did not enroll subjects with AHI <15 because CPAP compliance in these patients may not be optimal. For PSG, we scheduled bedtimes and morning awakening times to resemble each participant's usual habit. We made PSG recordings using Grass Heritage computerized polysomnographic systems. Briefly, standard surface electrodes were used to record electroencephalographic, electrooculographic, electromyographic (submentalis and anterior tibialis), and electrocardiographic activities. Nasal-oral thermocouples monitored airflow, while thoracic and abdominal movements indicated respiratory effort. The respiratory tracings were scored for the presence of apneas (10-second, or longer, cessation in nasal-oral airflow) or hypopneas (a 10-second, or longer, reduction of nasal-oral airflow of 30% or more with O_2 _desaturation more than 4% or arousal). Blood oxygen saturation was monitored with pulse oximetry. Recording and scoring technique followed the current American Academy of Sleep Medicine standards for human subjects. AHI was calculated to indicate the number of sleep-disordered breathing events/hour of sleep. Subjects qualifying for study underwent an oral glucose tolerance test (OGTT) and completed an Epworth Sleepiness Scale (ESS). After this baseline evaluation, the subjects underwent an attended CPAP titration with polysomnography. The best pressure was the one associated with the lowest AHI while the patient slept 20 minutes, or more. After titration, subjects received a CPAP machine and related accessories (Respironics, REMStar Pro) with card reader to monitor the compliance of CPAP and were followed for 6 months. Subjects were seen 2-3 times during the study and CPAP compliance was checked during the visits by using the EncorPro SmartCard (Respironics). CPAP efficacy was rechecked with overnight pulse oximetry at the end of the study. To mimic their real-life situation, subjects were given no specific instructions regarding diet or physical activity.

### Hormonal assays

Blood was collected in the morning between 7 and 8 AM in EDTA-containing tubes and kept at 4°C during processing. Aprotinin (100 μL containing 0.6 TIU per mL of blood) was added to one of the tubes and the samples were then centrifuged at 3000 rpm for 30 minutes. Active ghrelin levels were measured by a radioimmunoassay (RIA) kit (LINCO Research, St. Charles, MO) in plasma treated with HCL and phenylmethylsulfonyl-fluoride. Insulin and leptin levels were measured by a radioimmunoassay kit (Linco Research, St. Charles, MO) as we have previously described [9]. Glucose levels were measured in the same plasma samples by the MEDVAMC's laboratory. Adiponectin levels were measured by RIA with a kit from LINCO Research (St. Charles, MO) in diluted plasma samples (1:450). Resistin was measured in plasma samples by ELISA (Biovendor, Candler, NC).

### Oral glucose tolerance test (OGTT) and assessment of insulin sensitivity

The subjects underwent an early morning 75 g. OGTT at baseline and after six months of CPAP therapy. Blood samples were taken at -5, 30, 60, 90, and 120 min. for the measurement of plasma active ghrelin, glucose and insulin concentrations. Fasting insulin sensitivity was assessed using the homeostasis model assessment (HOMA) and the quantitative insulin sensitivity check index (QUICKI). Both HOMA [HOMA-IR = fasting glucose (mmol/L) × fasting insulin (microU/ml)/22.5] and QUICKI (1/[log fasting insulin + log fasting glucose]) were calculated as previously described. Estimates of insulin resistance from both indices correlate well with estimates from the "gold standard" hyperinsulinemic euglycemic clamp method [10,11]. In addition, from the OGTT we calculated a previously validated index of whole-body insulin sensitivity (ISI) (10,000/square root of [fasting glucose × fasting insulin] × [mean glucose × mean insulin during OGTT]), which is highly correlated (*r *= 0.73, p <0.0001) with the rate of whole-body glucose disposal during the euglycemic insulin clamp [12].

### Statistical Analysis

SPSS version 12.00 software for Windows (SPSS Inc. Chicago, IL) was used for statistical analysis. Parametric variables are expressed as mean ± S.E. unless otherwise stated. Categorical parameters are expressed as percentages. The areas under the curve (AUC_0-120_) for active ghrelin, insulin and glucose levels were calculated using the trapezoidal rule. For normally distributed data, statistical comparisons were performed using the Fisher's exact test or Chi-square test for categorical data and t-test for parametric data. Pearson's correlations were obtained between continuous variables. When data were not normally distributed, Wilcoxon rank test or Mann-Whitney tests were used and Spearman's correlation was obtained to measure associations between continuous variables. Linear regression tested the predictive value of changes in BMI and nadir SpO2 entered individually on the following outcomes: changes in insulin, insulin resistance as measured by HOMA-IR, leptin, ghrelin, adiponectin and resistin. Inclusion was set at probability *F*<0.05, and exclusion was set at *F*>0.10. Collinearity diagnostics used to test for multicollinearity included tolerance, variance inflation factor and condition index. Inferential analysis was conducted using an alpha error level of ≤0.05 to determine significance. Power calculations were done using paired t-test, two-sided methodology based on previously published insulin sensitivity and ghrelin mean changes from baseline where insulin sensitivity improved after 3 months of CPAP by 1.37 mcmol/Kg × min [13] and ghrelin decreased by 38.2 pg/mL after two days of CPAP [14] in OSA patients. Assuming a SD of 1.7 mcmol/Kg × min and 45 pg/mL respectively, we estimated that a sample size of 23 subjects would be sufficient to detect statistically significant differences (p ≤ 0.05) in the outcomes measured with a power of 90% and taking into account an attrition rate of 15% (20 completers).

## Results

Twenty-three subjects enrolled and 20 subjects completed the study. One subject died unexpectedly at home, from unknown cause. Two subjects were lost to follow up. We did not enroll any subjects with a diagnosis of diabetes. Table 1 shows demographic, PSG and metabolic parameters for these subjects.

**Table 1:** Baseline Subjects Characteristics (n = 20)

Age (yrs)	59.7 ± 2
Body weight (Kg)	108 ± 5.3

BMI (Kg/m^2^)	36.5 ± 1.8

Race (W, AA, H)	14, 4, 2

Male/Female	17/3

Leptin (ng/dL)	22.7 ± 6

Active ghrelin (pg/mL)	131 ± 48

Insulin (mU/mL)	22 ± 3

Adiponectin (ng/mL)	8.3 ± 1.2

Resistin (ng/mL)	3.1 ± 0.4

Glucose (mg/dL)	105 ± 4

QUICKI	0.31 ± 0.008

ISI	2.6 ± 0.55

HOMA-IR	5.9 ± 1

ESS	14.6 ± 1

AHI (episodes/hr)	50 ± 6

Lowest O2 sat. (%)	77 ± 3

Mean O2 sat. (%)	91.9 ± 0.9

Data shown are mean +/- SEM. BMI: Body mass index, W: White, AA: African American, H: Hispanic, QUICKI: quantitative insulin sensitivity check index, ISI: Insulin sensitivity index, HOMA-IR: homeostasis model assessment, ESS: Epworth Sleepiness Scale, AHI: Apnea/Hypopnea Index.

### Sleep parameters and CPAP compliance

CPAP effectively reversed hypoxia in all subjects (nadir O_2 _saturation 77 ± 3% at baseline and 89.3 ± 3 post CPAP, p = 0.005) although mean O_2 _saturation did not change significantly (Table 2). Subjects used CPAP for 165 ± 17 days and 5.3 ± 0.35 hrs/night. As shown in Table 2, ESS decreased with CPAP therapy. However, subjects as a group experienced weight gain after CPAP treatment compared to baseline with a mean difference of 1.6 Kg (p < 0.05) or 0.6 Kg/m^2 ^(p = 0.06). Systolic blood pressure, diastolic blood pressure and heart rate remained unchanged throughout the study period.

**Table 2:** Sleep and metabolic parameters before and after CPAP use

	Baseline	Post-CPAP	p value
CPAP pressure (cm H2O)		10 ± 3.2	

CPAP use (days)		165 ± 17	

CPAP use (Hrs/day)		5.3 ± 0.35	

ESS	14.6 ± 1	9.5 ± 1	**0.002**

Lowest O2 sat. (%)	77 ± 3	89.3 ± 3	**0.005**

Mean O2 sat. (%)	93.2 ± 0.7	93.8 ± 0.62	**0.5**

Systolic blood pressure (mmHg)	124 ± 3	129 ± 4	0.07

Diastolic blood pressure (mmHg)	76 ± 2	76 ± 2	0.99

Heart rate (bpm)	77 ± 3	72 ± 3	0.27

Body weight (Kg)	108 ± 5.3	109.6 ± 5.4	**0.04**

BMI (Kg/m^2^)	36.5 ± 1.8	37.1 ± 1.8	0.06

Leptin (ng/dL)	22.7 ± 6	21.6 ± 4	0.61

Adiponectin (ng/mL)	8.3 ± 1.2	8.2 ± 1.2	0.94

Resistin (ng/mL)	3.1 ± 0.4	3.2 ± 0.4	0.79

HOMA-IR	5.9 ± 1	7.5 ± 1.2	**0.04**

ISI	2.6 ± 0.55	2.1 ± 0.33	0.09

QUICKI	0.31 ± 0.008	0.3 ± 0.006	**0.02**

Significant differences compared to baseline (p ≤ 0.05) appear in **bold**. ESS: Epworth Sleepiness Scale, QUICKI: quantitative insulin sensitivity check index, ISI: Insulin sensitivity index, HOMA-IR: homeostasis model assessment.

### Glucose, insulin and insulin resistance

**Figure 1:**
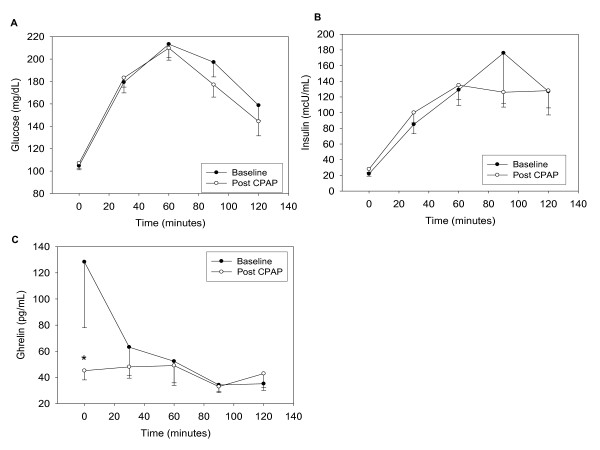
**Glucose (A), insulin (B) and active ghrelin levels (C) during OGTT before and after CPAP**. *p < 0.05 for baseline values. p values for fasting and AUC_0-120 _glucose were 0.88 and 0.24 respectively. p value for insulin AUC_0-120 _was 0.7; p value for ghrelin AUC_0-120 _was 0.4.

Fasting and postprandial glucose levels were unchanged after CPAP use compared to baseline (Figure 1A). Fasting insulin levels increased significantly after CPAP use (Figure 1B). However postprandial and AUC_0-120 _insulin remained unchanged compared to baseline (baseline insulin AUC_0-120 _549 ± 129 μU*h/mL, post-CPAP insulin AUC_0-120 _491 ± 56 μU*h/mL; p = 0.7). Insulin resistance increased as measured by HOMA-IR, QUICKI and ISI, although it only reached significance for the first two indices (Table 2).

### Active ghrelin and adipokine levels

Fasting active ghrelin levels decreased significantly after CPAP use. However, postprandial active ghrelin levels and active ghrelin AUC_0-120 _remained unchanged compared to baseline (Figure 1C). Circulating leptin, adiponectin and resistin levels remained unchanged after CPAP use (Table 2).

### Correlation and regression analyses between changes in body weight, hormones and sleep parameters

Changes in BMI were directly correlated with changes in insulin levels and in insulin resistance as measured by HOMA-IR. Changes in ghrelin levels were inversely correlated with changes in insulin resistance, although there was no correlation between changes in ghrelin and changes in BMI or any of the other parameters measured (Table 3). On regression analyses, changes in BMI predicted changes in insulin (B = 4.9 ± 2, p = 0.03), insulin resistance (B = 1.75 ± 0.65, p = 0.02) and leptin (B = 2.2 ± 1, p = 0.046) but not on ghrelin (B = 38 ± 72, p = 0.61), adiponectin (B = -0.02 ± 1, p = 0.98) or resistin (B = -0.09 ± 0.25, p = 0.74). Nadir SpO2 did not predict any of the outcome variables (B = 0.8 ± 0.78, p = 0.78 for insulin; B = 0.15 ± 0.25, p = 0.6 for HOMA-IR; B = 0.46 ± 0.39, p = 0.26 for leptin; B = -0.36 ± 24, p = 0.17 for ghrelin; B = 0.34 ± 0.42, p = 0.44 for adiponectin and B = -0.09 ± 0.095, p = 0.37 for resistin). Baseline AHI correlated with changes in ESS (r -0.57, p 0.009) but was not correlated with CPAP use, changes in nadir or mean O_2 _or any of the other metabolic parameters. Baseline ESS did not correlate with baseline HOMA-IR.

**Table 3:** Correlation analysis for changes in weight, hormone levels and sleep parameters [r(pvalue)]

	HOMA- IR	Ghrelin	Leptin	Insulin	Adiponectin nectin	Resistin	ESS	CPAP use
BMI	**0.56** **(0.01)**	0.04 (0.87)	0.32 (0.17)	**0.58** **(0.008)**	-0.24 (0.29)	-0.35 (0.13)	-0.02 (0.95)	0.02 (0.94)

HOMA- IR		**-0.51** **(0.026)**	0.13 (0.59)	**0.95** **(0.001)**	-0.04 (0.9)	-0.32 (0.18)	-0.01 (0.98)	0.22 (0.35)

Ghrelin			0.18 (0.46)	-0.43 (0.066)	-0.27 (0.26)	0.11 (0.68)	0.09 (0.7)	-0.19 (0.43)

Leptin				0.22 (0.34)	0.21 (0.38)	0.11 (0.65)	0.15 (0.53)	-0.27 (0.26)

Insulin					-0.17 (0.48)	0.4 (0.08)	-0.07 (0.8)	0.21 (0.37)

Adipon ectin						0.24 (0.33)	0.13 (0.59)	-0.11 (0.65)

Resistin							0.04 (0.87)	-0.26 (0.28)

Significant correlations (p ≤ 0.05) appear in **bold**. ISI: Insulin sensitivity index, HOMA-IR: homeostasis model assessment. Changes in all variables including ESS were use for analysis.

### Subgroup analyses

**Figure 2:**
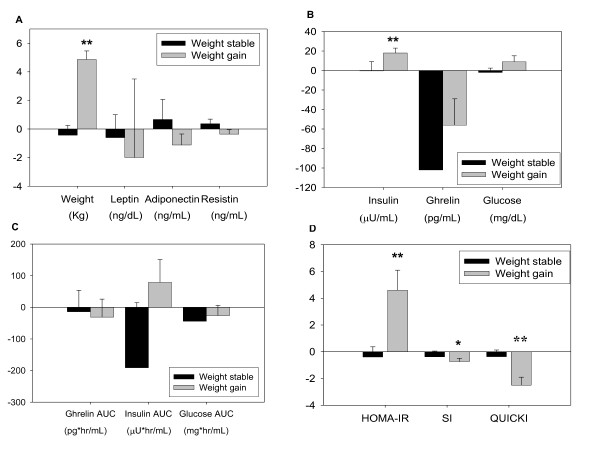
**Body weight, adipokines (A), glucose, insulin (B-C) and insulin resistance changes (D) after CPAP according to changes in body weight**. Weight gain was defined as an increase ≥2% of their initial body weight (n = 8). Weight stable was defined as a weight fluctuation ≥2% (n = 12). *p < 0.05, **p < 0.01 compared to other group.

To determine the effect of weight changes in the other parameters measured, we analyzed separately the data from those subjects who gained a significant amount of weight (defined as an increase ≥2% of their initial body weight, n = 8) and those whose body weight remained stable (n = 12). There were no significant differences at baseline between the two groups and none of the groups experienced significant changes in blood pressure or heart rate (data not shown). Leptin, resistin and adiponectin levels after CPAP remained stable in both groups compared to baseline (Figure 2A).

Fasting insulin levels were significantly increased in subjects who experienced weight gain but remained stable in those subjects with stable body weight. Fasting glucose levels remained unchanged in weight stable individuals and tended to increase in subjects experiencing weight gain but it did not reach statistical significance (Figure 2B). Postprandial and AUC_0-120 _insulin and glucose levels remained unchanged in both groups after CPAP use (Figure 2C). Fasting ghrelin levels decreased in both groups; although it did not reach statistical significance. Insulin resistance as measured by HOMA-IR, ISI and QUICKI remained unchanged in subjects with stable body weight. However, it was significantly increased in the weight gain group (Figure 2D).

## Discussion

Our study suggests that glucose metabolism is disturbed in obese patients with OSA and that weight change rather than hypoxia is the major long-term modulating factor in insulin resistance after CPAP treatment in this population. These findings also suggest that CPAP alone may not reduce body weight, and that in the face of weight gain CPAP treatment may not reduce insulin resistance and leptin or increase adiponectin in obese subjects. The results of our regression analyses where the predictive value of BMI and nadir SpO2 was explored support this hypothesis given that changes in BMI but not changes in nadir SpO2 predicted changes in insulin, insulin resistance and leptin.

We did not observe any changes in blood pressure, or heart rate after CPAP treatment in contrast to what most [15-17] but not all studies [18-20] have reported. Possible explanations for this discrepancy include: 1) A higher body weight in our cohort compared to others or the fact that body weight remained stable or increased in our cohort. This could have negated the beneficial effects of CPAP on these outcomes as suggested by a previous report that showed that the course of hypertension in OSA is more closely linked to weight loss than to elimination of sleep apnea by CPAP [16]; 2) Different duration of CPAP treatment (6 months in our study v. 1-2 months in other reports); 3) Time of the day at which BP was assessed given that CPAP effects on BP are reportedly more pronounced during sleep and we monitored our patients in the morning; 4) Methods of BP measurement since this factor has been shown to influence results [17]; and 5) We did not power the study to detect differences in these outcomes so a negative result should be interpreted with caution.

Several reports have demonstrated an association between OSA and insulin resistance [2,21-24]. However, the effect of CPAP therapy on insulin resistance remains controversial (recently reviewed in [25]). Some reports failed to detect an improvement in insulin sensitivity [26], others showed an improvement in glucose levels only during sleep [27,28] and others showed an almost immediate improvement, especially in non-obese patients [13]. In our study, we found increased insulin resistance after 6 months of CPAP use. This insulin resistance was associated with weight gain indicating that body weight plays a major role in determining insulin resistance in obese CPAP-treated patients with OSA. These results are in agreement with those reported by Ip and others [21]. The apparently divergent findings between our results and those previously reporting an improvement in insulin sensitivity also may relate to differences in sample timing. Our assessment was done 6 months after starting treatment whereas most reports have been done between 48 hours and 3 months after starting CPAP. It is possible that CPAP use has only a transient effect on insulin sensitivity and that changes in body weight are a much more important factor in the long-term regulation of insulin sensitivity.

Ghrelin is an appetite-increasing hormone postulated as a contributor to OSA-associated obesity as ghrelin levels were elevated in one report [14]. In the same study, fasting total (the sum of active and inactive) ghrelin levels decreased after 2 days of CPAP. Another study reported equivalent fasting total ghrelin levels in obese subjects with OSA and BMI matched controls without OSA [29]. In our study, we measured active ghrelin instead of total ghrelin because 75% of the circulating peptide is biologically inactive and the ratio between inactive and active ghrelin changes in different clinical scenarios [9]. Since ghrelin is suppressed by food intake, ghrelin levels were measured while fasting and during the OGTT. Our results show that 6 months of CPAP treatment significantly decreased fasting active ghrelin levels but that postprandial levels of this hormone remained unchanged. This is in agreement with a recent report of fasting active ghrelin levels being decreased by CPAP after one month of treatment [30]. Although ghrelin inversely correlates with body weight in the setting of obesity, we did not found any association between changes in ghrelin levels and changes in BMI, CPAP use or changes in the ESS in this setting. Ghrelin correlated with changes in insulin resistance, suggesting that other factors besides body weight may play a role in its regulation including changes in insulin sensitivity. Insulin administration has been shown to suppress circulating ghrelin levels in some [31] but not all studies [32]. Plasma insulin levels and insulin resistance correlate inversely with ghrelin. This association was BMI-independent in some studies [33]. However in a study using euglycemic hyperinsulinemic clamp method, insulin sensitivity did not correlate with ghrelin concentrations [34]. Independent of metabolic factors, ghrelin may also act as a sleep-inducing hormone. Ghrelin levels increase after sleep deprivation [35] and slow wave sleep is enhanced after ghrelin administration [36]. Based on these data, we postulate that the fasting ghrelin level increase seen in patients with OSA is a compensatory response to poor-quality sleep and could explain why fasting ghrelin levels decreased after CPAP use.

Leptin is secreted by adipocytes in proportion to body fat, being elevated in obese individuals and decreasing with weight loss. Leptin-deficient animals exhibit respiratory depression and CO_2 _retention. Leptin administration to these animals increases minute ventilation and improves lung mechanics [37]. These animal experiments suggest that an increase in leptin levels in patients with OSA may represent a compensatory response to hypoxia. Consistent with this hypothesis, elevated leptin has been described in OSA patients compared to BMI-matched controls. This elevation in leptin was reversed by CPAP treatment [14,38], although this was associated with a decrease in fat accumulation in some studies [39] that may have accounted at least partially for the changes in leptin. Others have reported that leptin levels are similar in obese OSA patients when compared to non-OSA controls and that these levels do not change significantly after 1 month or 1 year of CPAP [30,40]. In agreement with the latter study, our data showed that leptin levels remained stable after CPAP use. Taken together, these data suggest that if CPAP has an effect on leptin levels, it is short-lasting.

The role of resistin in diabetes remains a matter of debate. Circulating resistin levels directly correlate with BMI and have been shown to decrease with weight loss [41]. Resistin also directly correlates with insulin resistance in some studies, but not in others [42,43]. In our study, resistin levels did not change after 6 months of CPAP and its levels did not correlate with changes in body weight, insulin and other adipokines or sleep parameters. In agreement with our data, resistin levels were stable after 2 days and 2 months of CPAP use in a group of subjects with OSA, suggesting that resistin is unlikely to play an important role in the insulin resistance or obesity seen in OSA [13].

Adiponectin is decreased in obese individuals and in those with type 2 diabetes. It is thought to play a role in many of the metabolic complications suffered by these patients including metabolic syndrome and cardiovascular disease. However, its role in patients with OSA remains controversial. Elevated adiponectin was found in subjects with OSA when compared with non-OSA controls in one report and diminished in another [44,45]. In agreement with prior reports of adiponectin levels after CPAP use [46], we report here that adiponectin levels remained unchanged after 6 months of CPAP treatment. Harsch et al. had previously reported a decrease in adiponectin levels after 48 hrs of CPAP use but levels returned to baseline at 3 months. The data suggest that chronic CPAP treatment does not play a role in the regulation of adiponectin levels.

Although the study was powered a priori using published data [13,14], the small sample size is a limitation of this study. Other limitations include the lack of data on changes in dietary habits; physical activity and body composition that could help us better understand the effects of CPAP on hormonal regulation. Also, it would have been useful to compare changes in body weight and other parameters with a non-interventional group of controls. However, such a group was not included in our design because these subjects have a clinical indication for CPAP use and delaying its use would have been unethical. Our study was powered to detect significant differences in insulin resistance and ghrelin levels. Consequently, we cannot conclude that the lack of changes in leptin, adiponectin and resistin levels in this relatively small sample would not be seen in a larger sample. Significant associations detected during simple correlation analysis should be interpreted with caution given the number of variables compared which increase the chance for a type I error. Future studies should include a larger number of patients along with an assessment of dietary habits; physical activity, energy expenditure, anthropometrics (i.e. waist-to-hip ratio) and body composition in order to better understand the effects of CPAP in this setting.

## Conclusions

In summary, six months of CPAP treatment did not improve insulin resistance in obese subjects. In fact, in subjects who gained weight during the study, insulin resistance increased suggesting that changes in insulin sensitivity induced by CPAP in this setting are mainly determined by changes in body weight. CPAP treatment induced a decrease in fasting ghrelin levels, although body weight increased in most subjects. Adipokines such as leptin, adiponectin and resistin also appear to be influenced much more by adiposity rather than hypoxia. The fact that these adipokines remain unchanged after 6 months of CPAP treatment suggests that they are unlikely to play an important role in the development of the metabolic complications seen in the setting of OSA. When obese patients with OSA are treated with CPAP, other measurements targeting obesity should also be pursued.

## Abbreviations

AHI: Apnea+Hypopnea Index; CPAP: Continuous Positive Airway Pressure; ESS: Epworth Sleepiness Scale; HOMA: Homeostasis Model Assessment; IR: Insulin Resistance; ISI: Insulin Sensitivity Index; OGTT: Oral Glucose Tolerance Test; PSG: Polysomonography; QUICKI: Quantitative Insulin Sensitivity Check Index; RIA: Radioimmuniassay.

## Competing interests

The authors declare that they have no competing interests.

## Authors' contributions

JMG and AS participated in the design of the study and in writing the manuscript. HS recruited patients and collected the data. MH and RN performed the PSG studies. JG performed the statistical analysis and hormonal assays. JG, HS, RN, MH, AS reviewed and approved the final version of the manuscript.
